# Melatonin Hormone Acts on Cells of Maternal Blood and Placenta From Diabetic Mothers

**DOI:** 10.3389/fphys.2021.765928

**Published:** 2022-01-21

**Authors:** Martino B. Pierre Louis, Danielle Cristina Honorio França, Adriele Athaídes Queiroz, Iracema de Mattos Paranhos Calderon, Eduardo Luzía França, Adenilda Cristina Honorio-França

**Affiliations:** ^1^Institute of Biological and Health Science, Federal University of Mato Grosso, Barra do Garças, Brazil; ^2^Medical Course, State University of Mato Grosso, Cáceres, Brazil; ^3^Gynecology, Obstetrics and Mastology Posgraduate Programme, Botucatu Medical School, São Paulo State University (UNESP), Botucatu, Brazil

**Keywords:** apoptosis, mononuclear cells, diabetes, melatonin, placenta, blood

## Abstract

Changes in glucose metabolism of diabetic mothers affect immunological components, proinflammatory factors, and placental hypervascularization that can induce cell death. The hormone melatonin has been identified as a potential modulating agent. The aim of this study was to analyze the oxidative process and the apoptosis in maternal blood and placental cells modulated by melatonin from diabetic mothers. The groups were 40 pregnant women divided into non-diabetic (ND) and type 2 diabetes mellitus (T2DM) groups. Blood and placental cells were obtained by density gradient and maintained in culture treated or not with melatonin (100 ng/mL) for 24 h (37°C, 5% CO_2_). Oxidative stress was evaluated by superoxide release and CuZn superoxide dismutase (SOD). Apoptosis was assessed by flow cytometry. Maternal hyperglycemia increased superoxide release and apoptosis in MN cells from maternal blood and reduced SOD level and SOD/O2- ratio. Melatonin reduced oxidative stress and apoptosis rates in MN cells in the blood of diabetic mothers. There was a reduction in SOD and SOD/O2- ratio in the placental extravillous layer, and melatonin restored the concentrations of this enzyme. There was greater superoxide release, reduced SOD/O2- ratio, and apoptosis in MN cells placental villous layer. Melatonin increased apoptosis rates in the placental villous layer from hyperglycemic mothers. These data suggest that hyperglycemia altered the processes oxidative in blood and placenta from hyperglycemic mothers. These changes reflected in the mechanisms of induction of apoptosis, especially in the vascularized layers of the placenta, and were modulated by melatonin.

## Introduction

The placenta is a highly vascularized transitory organ from fetal–maternal tissues that play important functions in maintaining pregnancy and promoting fetal development (Moore and Persaud, 2008). This organ plays a critical role in immunoregulation (Hara et al., 2016) and sources cells from maternal and fetal origin. These cells present molecules that have an essential role in maternal–fetal tolerance (Guleria and Sayegh, 2007).

The placenta can alter cell and cytokine levels from maternal blood before passing them on to the fetus. The inflammatory environment influences this mechanism due to hyperglycemia (Hara et al., 2016) that causes many major complications macro-and microvascular damage (American Diabetes Association [ADA], 2014).

Maternal hyperglycemia may trigger structural and physiological responses to assure maternal–fetal exchanges and fetal oxygen delivery and alter cellular oxidative metabolism at the maternal–fetal interface (Gauster et al., 2011) with an imbalance between the generation of reactive oxygen species (ROS) and antioxidant defense.

These changes cause modification of cellular proteins, lipids, and DNA, affecting cellular behavior and differentiation and inducing apoptosis and cellular damage (Espinosa-Diez et al., 2015). In addition, in the placenta villous layer of diabetic mothers, memory T cells and Fas expression are reduced, which may alter T cell apoptosis and regulate maternal–fetal immune tolerance (Queiroz et al., 2019).

The placenta, through maternal blood, establishes an interface for the exchange of nutrients and gases with the fetus, and the production of specific regulatory molecules with metabolic and endocrine activities, with the participation of several hormones (Benirschke, 2000). For example, the hormone melatonin has effects on pancreatic insulin secretion (Fu et al., 2013) and cellular oxidative metabolism (Fernandes et al., 2019), and antioxidant action (Arendt and Skene, 2005; Morceli et al., 2013).

There is evidence of the importance of melatonin as a potent immunomodulatory agent that improves the functional activity of cells (França et al., 2008; Honorio-França et al., 2009, Honorio-França et al., 2013). In addition, melatonin can bind to phagocytes and trigger various oxidative processes in the body (Fernandes et al., 2019).

It is known that melatonin stimulates the release of active oxygen metabolites by immune cells (Honorio-França et al., 2013). The mechanisms by which melatonin influences immune functions involve the participation of other hormones, cytokines, and specific receptors (Pandi-Perumal et al., 2008). Studies with macrophages in experimental models of diabetes and diabetic patients report that melatonin exerts antioxidant action. In animal cells and non-diabetic individuals, it has a pro-oxidative effect (França et al., 2008; Honorio-França et al., 2009; Morceli et al., 2013). However, the effects of melatonin on the oxidative process on placental cells and their action on the maternal–fetal interface have not yet been elucidated. Melatonin may act in the cellular oxidative process by favoring the development of the fetus and the vascularization of the uteroplacental in pregnancies of diabetic mothers. Thus, this study proposed to analyze the cellular oxidative process and the induction of apoptosis in maternal blood and placental cells modulated by melatonin from diabetic mothers.

## Materials and Methods

A cross-sectional study was performed with blood and placenta from diabetic mothers. The mothers were attended the Diabetes and Pregnancy Facility, School of Medicine Obstetrics Course, UNESP, Botucatu, SP. The local Research Ethics Committee approved this study, and all the women gave informed written consent.

### Subjects

For analysis of blood and placenta, the pregnant women (18–45 years old) were separated by maternal glycemic status. Pregnant women with diabetes mellitus type 2 (T2DM) were referred to the service with a confirmed diagnosis. The non-diabetic pregnant women were evaluated by a 75-g oral glucose tolerance test (OGTT-75 g-American Diabetes Association [ADA], 2014) and glucose profile (GP; Rudge et al., 2000; Calderon et al., 2007) between the 24th and 28th weeks of pregnancy. Thus, according to the test results, 40 pregnant women were classified into the following groups: the non-diabetic (ND) group (normal 75 g-OGTT and normal GP; *n* = 20) and the type 2 diabetes mellitus (T2DM) group (abnormal GTT-75 g, prior to pregnancy *n* = 20) (American Diabetes Association [ADA], 2014).

Independent of diagnosis, the pregnant women continued attending the service. Patients with T2DM were evaluated every 2 weeks until the 32nd week for GP with fasting, pre-and post-prandial glycemic levels for 24 h. In addition, they were treated with physical exercise, a specific diet, and insulin therapy from the beginning of the pregnancy until delivery (Calderon et al., 2007) for glycemic control (Rudge et al., 2000). A glycaemic mean of 120 mg/dl or less was defined as adequate glycaemic control, and a glycaemic mean higher than 120 mg/dL was defined as inadequate glycaemic control. The ND pregnant women did not receive any type of therapy for glycemic control.

These patients were individualized by gestational age at delivery, altered blood pressure, and body mass indexes. In addition, women with gestational age until 20 weeks who received prenatal and delivery care at the Service and signed a Consent Form and delivered in the morning were considered inclusion criteria. The exclusion criteria were women with multiple pregnancies, T1DM, GDM, fetal malformations, and deliveries before the 34th week of gestation.

### Blood Sampling and Separation of Blood Cells

Before labor, approximately 8 mL of blood sample was collected into heparinized (25 U/ml) tubes in the morning (8:00 at 10:00 h) at the 36th week of pregnancy. The plasma was retired and maintained at −80°C for the determination of glucose, melatonin, and SOD. The cells were obtained by fractionated with Ficoll-Paque density gradient (density 1.077 g/L; centrifugation 160 × *g*; 40 min; Pharmacia, Upsala, Sweden) and resuspended independently in serum-free medium 199 at a final concentration of 2 × 106 cells mL^–1^ and used immediately for assays of superoxide release and apoptosis.

### Placenta Sampling and Separation of Cells

Placenta was obtained at delivery and washed with saline solution. A sample was collected along the placenta to remove the villous region with a margin of approximately 2 cm from the insertion of the umbilical cord, midway between the maternal and fetal sides. The large vessels were removed, leaving only the villous tissue (22,23). The basal plate was carefully dissected from the villous tissue and amniochorium membrane. The isolation of extravillous tissue was adapted from the procedure described for isolating amniochoric cytotrophoblast and removing the extravillous region from the placental border (Calderon et al., 2007; Stenqvist et al., 2008; Vincent et al., 2015). The fragments were stored in liquid nitrogen for later obtaining of cells. The samples (100 mg of tissue/ml) were macerated in PBS with Tween 20 supplemented with protease inhibitors (0.1 mM phenylmethylsulfonyl fluoride; 0.1 mM benzethonium chloride, 10 mM EDTA, 20 UI aprotinin, and 0.5% BSA) using a homogenizer Power Gen 125 (Fisher Scientific^@^). The homogenate was filtered and reserved for MLT and SOD determination, and the sediment (cells) was fractioned by Ficoll-Paque gradient (density 1.077 g/L; centrifugation 160 × *g*; 40 min; Pharmacia, Upsala, Sweden). After centrifugation, the cells were collected using a siliconized Pasteur pipet and transferred to tubes. The cells were washed twice with Medium 199 (Sigma Chemical, St. Louis, MO, United States) for superoxide release and apoptosis assays.

### Glucose Determination

Glucose levels were quantified by the glucose oxidase method (Glucose – analyzer II Beckman, Fullerton, CA, United States). HbA1c was determined by high-performance liquid chromatography (D10*™* hemoglobin testing system, BIO-RAD Laboratories, Hercules, CA, United States).

### Determination of Melatonin

The hormone melatonin (plasma and placenta homogenate) was quantified by the Melatonin ELISA kit (IBLHamburg, German). The kit has the following characteristics: the lower detection limit was 1.6 pg/ml, and intra-assay and inter-assay coefficients of variation (%) were 3.0–11.4 and 6.4–19.3, respectively. Melatonin extraction was performed by the affinity chromatography method using standardized columns. Columns were placed in glass tubes and centrifuged twice with 1 ml methanol (1 min – 200 × *g*). Then the columns were washed two times with double distilled water (1 min – 200 × *g*). After preparation of columns, 0.5 ml of standards, controls, and samples were applied and centrifuged for 1 min at 200 × *g*. After applying the samples and standards, the columns were washed again with 1.0 ml of 10% methanol for 1 min at 500 × *g*. Next, the extraction of the eluate containing the hormone melatonin was carried out by adding 1.0 ml of methanol at 200 × *g*. After obtaining the eluate, the methanol was evaporated using an evaporator centrifuge (speed-vac). The material was reconstituted with 0.15 ml of double distilled water under stirring for 1 min and immediately analyzed. 50 ml of each standard, control, and colostrum and milk samples were placed in an ELISA plate with 50 ml of melatonin-biotin in each well with 50 ml of antiserum. The plate was incubated at 4°C for 20 h. After this period, the supernatant was discarded, the plate was washed 3 times with wash buffer, and 150 ml of the conjugated enzyme was added. After 120 min of incubation at room temperature, the plate was washed 3 times, 200 ml of the substrate p-nitrophenyl phosphatase (PNPP), and incubated for another 40 min under agitation. After this period, the reaction was blocked with 50 ml of “PNPP stop” solution, and the reading was done in a spectrophotometer for a 405 nm filter. Results were calculated using the standard curve (*R*^2^ = 0,984) and expressed in pg/mL (Honorio-França et al., 2013).

### Cells Treatment

Blood and placental MN cells treated or not with 50 μL of melatonin (MLT −100 ng/mL final concentration- Honorio-França et al., 2013) were incubated for 24 h (37°C; 5% CO_2_). After the cells were resuspended in RPMI 1640 medium containing 10% fetal bovine serum (FBS-Sigma, St. Louis, MO, United States), the cells were tested for superoxide release and apoptosis. The culture supernatant was reserved for quantitation of the superoxide enzyme (SOD). For each assay performed, a phagocyte control (2 × 10^6^ cells/ml) was incubated for a similar time, depending on the type of essay in medium 199 or PBS, in the absence of melatonin.

### Superoxide Anion Determination

Superoxide release was evaluated by method reduction of cytochrome C (Sigma, St Louis, MO, United States; Honorio-França et al., 1997). Blood and placental MN cells treated or not with MLT were incubated for 24 h. After this time, the cells were centrifuged (160 × *g*, 10 min) and resuspended in PBS containing 2.6 mM CaCl_2_, 2 mM MgCl_2_, and cytochrome C. The cells (100 μL) were then incubated on culture plates at 37°C for 1 h. A control of untreated cells was used to evaluate spontaneous release. The reactions were measured by absorbance at 550 nm, and the results are expressed as nmol. O_2_^–^. The experiments were performed in duplicate.

### CuZn-Superoxide Dismutase Determination (CuZn-Superoxide Dismutase–E.C.1.15.1.1)

Analysis of the CuZn-SOD enzyme was determined in plasma, placenta homogenate, and cell culture supernatants treated or not with melatonin from maternal blood and placenta using the nitroblue tetrazolium (NBT) reduction method (Sigma, St Louis, MO, United States; Novelli et al., 1993). The individual samples (0.5 mL) and standard (hydro-alcoholic solution) were placed in glass tubes. Next, 0.5 mL of chloroform-ethanol solution (1:1 ratio), 0.5 mL of reactive mixture (NBT and EDTA) and 2.0 mL of buffer carbonate/hydroxylamine (*pH* = 10.2) were added. The tubes remained still at room temperature for 15 min and were subsequently read at 560 nm. The results were expressed in international units (IU) of CuZn-SOD and were calculated by the following equation:


$$\begin{array}{c} \text{CuZn-SOD}=\left(\mathrm{Ab}\mathrm{standard}-\mathrm{Ab}\mathrm{sample}/\mathrm{Ab}\mathrm{standard}\right)\\ \;\times 100=\%\mathrm{reduction}\text{of}\mathrm{NBT}/\mathrm{CuZn}\text{-SOD}. \end{array}$$

### Apoptosis Assay

To determine the apoptosis rates, and APO-DIRECT*™* kit (BD Biosciences - United States) was used. As per the manufacturer’s instructions, apoptosis tests were performed. The flow cytometry (FACSCalibur system; BD, San Jose, United States) was used to analyze the results, and the data were evaluated by Cell Quest software.

### Statistical Analysis

The Student’s *t*-test was used to evaluate the age, gestational age, BMI, placental weight and index, glucose, glycated hemoglobin, melatonin, and superoxide dismutase. The chi-square test analyzed qualitative variables (hypertension, smoking, and physical exercise). Analysis of variance (one-way ANOVA) and Tukey’s tests were used to evaluate the superoxide release, superoxide dismutase (in culture), and apoptosis index of MN cells from blood and placenta treated or not with melatonin. Statistical significance was considered when *p* < 0.05.

## Results

Clinical data of the mothers (ND and T2DM) are shown in Table 1. Pregnant women showed similarities in gestational age at birth, maternal age, and weight before pregnancy. Glycated hemoglobin levels were increased in diabetic mothers (Table 1).

**TABLE 1:** Clinical data on the pregnant women non-diabetic (ND) and T2DM (type 2 diabetes mellitus).

Parameters	ND	T2DM
Age (years)	27,1 ± 3.8	29.5 ± 5.3
Gestacional Age (weeks)	38,0 ± 1.2	37.8 ± 0.8
Glucose level (mmol/L)	4,2 ± 0.7	5.9 ± 0.8*
HbA1c (%)	5,2 ± 0.4	6.4 ± 0.8*
BMI-1	27,2 ± 4.2	30.3 ± 6.0
BMI-2	31,8 ± 7.3	34.4 ± 8.9
Hypertension	10%	40%^#^
Smoking	10%	5%^#^
Physical exercise	25%	70%^#^
Placental weight (g)	612.5 ± 99.4	716.6 ± 112.6
Placental Index	0.162 ± 0.035	0.179 ± 0.031*

*HbA1c - Glycated hemoglobin; BMI-1 and BMI-2 (body mass index in the first and third trimesters of pregnancy, respectively).*

*Data correspond to the median of 40 mothers.*

*The placental index is the ratio of placental weight to fetal weight.*

*P < 0.05 statistical difference (Student’s t-test).

*^#^P < 0.05 statistical difference (Chi-square test).*

**FIGURE 1:**
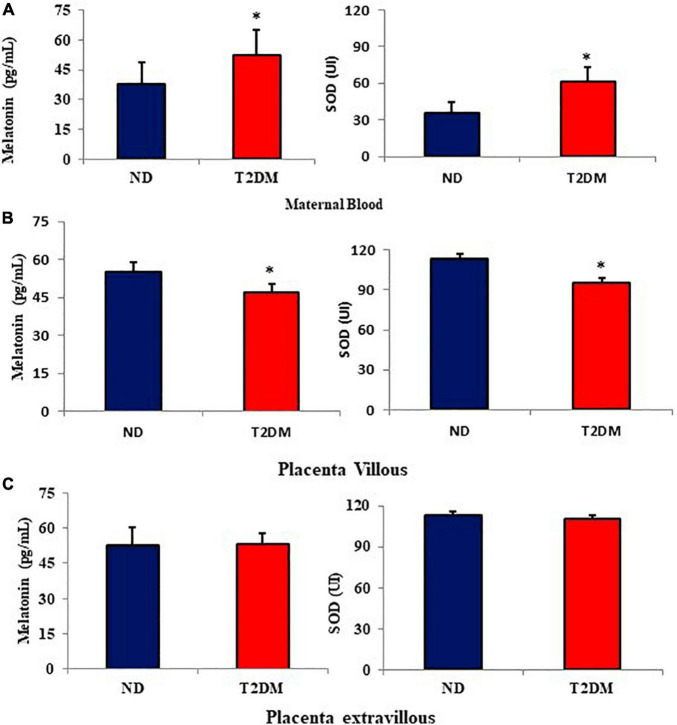
Mean (±SD; *N* = 10) melatonin levels (pg/mL) and superoxide dismutase (SOD- UI) in the maternal blood **(A)**, placenta villous **(B)**, and placenta extravillous **(C)** from diabetic mothers. *P < 0.05 (Student’s *t*-test) indicates the difference between normoglycemic and hyperglycemic groups.

The melatonin and superoxide dismutase (SOD) levels were evaluated in both groups’ maternal blood and placenta (Figure 1). The melatonin and SOD levels were higher in the maternal blood from diabetic mothers (Figure 1A). In placenta villi, both melatonin and SOD were lower in the diabetic group than in the ND group (Figure 1B). In contrast, in the placental extravillous layer, melatonin and SOD were similar among the groups (Figure 1C).

In culture supernatants from maternal blood MN cells, the SOD levels were lower in the diabetic group. Similar concentrations of the enzyme were observed in cells treated with melatonin in both studied groups. Similar SOD levels were later observed in MN cells in the placenta villi, irrespective of melatonin treatment. A reduction in SOD levels was observed in the MN cell culture supernatants from the placenta extravillous layer of diabetic mothers compared to ND mothers. Similar enzyme values were found in the culture supernatants when these cells were incubated with melatonin, regardless of the glycemic level of the study subjects (Table 2).

**TABLE 2:** Superoxide dismutase (SOD) levels in the culture supernatant of maternal blood cells, placenta villi, and placenta extravilli treated or not with melatonin from diabetic mothers.

SOD (UI)		ND	T2DM
Maternal blood	MLT (−)	14.6 ± 6.3	4.71 ± 1.2*
	MLT (+)	17.7 ± 7.9	18.2 ± 6.1^#^
Placenta villous	MLT (−)	18.3 ± 5.9	17.2 ± 3.4
	MLT (+)	15.8 ± 7.1	13. ± 6.8
Placenta extravillous	MLT (−)	20.2 ± 5.4	9.3 ± 5.7*^†^
	MLT (+)	21.9 ± 8.9	19.2 ± 8.3^#^

*The results represent the mean and standard deviation of 10 samples.*

*MN, Mononuclear cells (MN); MLT, melatonin.*

P < 0.05 statistical difference (ANOVA test) * comparing the groups considering the same treatment and type of sample; ^#^ comparing untreated cells with cells treated with melatonin, considering the same group and sample; ^†^comparing between villous and extravillous placental layers considering the same treatment and group.

**FIGURE 2:**
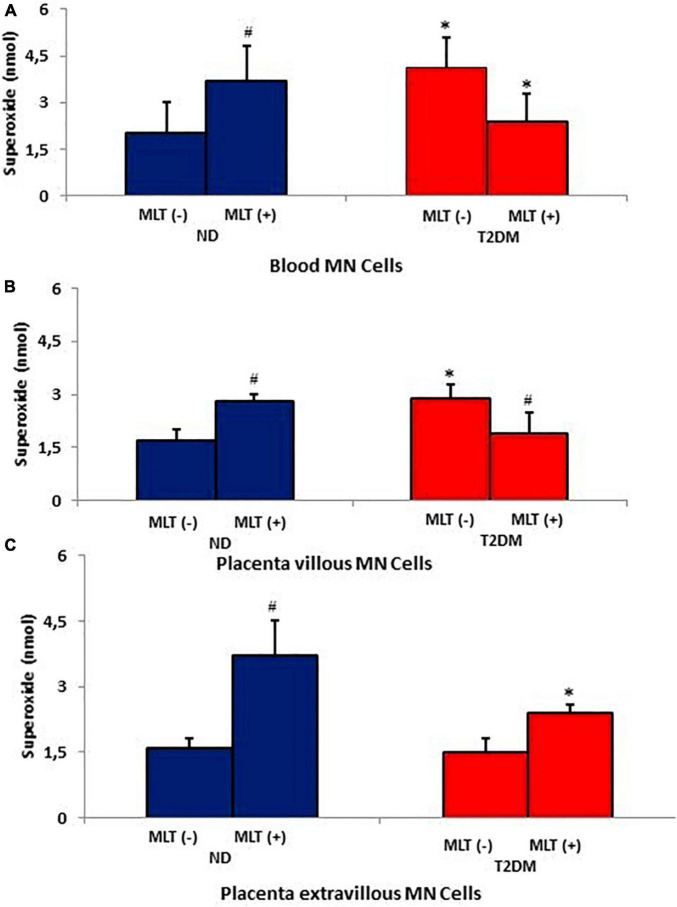
Release of superoxide (O2-) by maternal blood phagocytes **(A)**, villous placenta **(B)**, and extravillous placenta **(C)** treated or not with melatonin from diabetic pregnant women. The results represent the mean and standard deviation of 10 MN cell samples from different mothers in each group. *P* < 0.05 statistical difference (ANOVA) * comparing the groups, considering the same treatment and type of sample; ^#^*p* < 0.05, comparing cells treated with untreated melatonin, considering the same group and sample. Non-diabetic (ND); Type 2 Diabetes Mellitus (T2DM); melatonin (MLT); mononuclear cells (MN).

Superoxide release was higher in MN cells from maternal blood from diabetic mothers than in MN cells from ND mothers. In the non-diabetic group, blood MN cells stimulated with melatonin presented higher superoxide release than untreated cells (*p* < 0.05). However, melatonin did not increase superoxide release in blood MN cells in the hyperglycemic group (Figure 2A).

Irrespective of placental section, superoxide release was higher in MN cells from the placental villous layer of diabetic mothers. Melatonin increased superoxide release in cells from the placental villous layer (Figure 2B). However, superoxide release was lower in MN cells from placenta extravillous from the T2DM group (Figure 2C). The highest superoxide levels were observed in phagocytes of hormone-treated non-diabetic mothers (Figure 2C).

**FIGURE 3:**
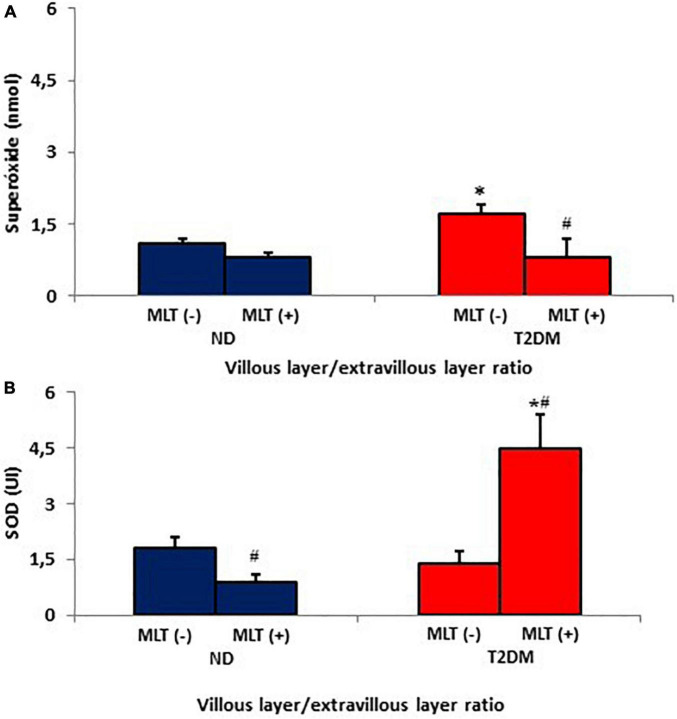
Villous placenta/extravillous placenta ratios from the release of superoxide **(A)** and superoxide dismutase enzyme (SOD) **(B)** by MN cells of the non-diabetic (ND) and type II diabetes mellitus (T2DM) groups. The results represent the average and standard deviation of 10 samples. *P* < 0.05 statistical difference (ANOVA) * comparing groups considering the same treatment; ^#^*p* < 0.05, comparing untreated cells with cells treated with melatonin, considering the same group.

The placenta villous/extravillous superoxide release ratio is shown in Figure 3A. It was observed that there was an increase in the anion release ratio between the MN cells of the different placental layers in the diabetic group compared to the ND group. In contrast, melatonin treatment reduced the placenta villous/extravillous superoxide ratio.

Figure 3B shows the placenta villous/extravillous SOD ratios. There was a reduction of the enzyme in the placenta villous/extravillous ratio in the diabetic group compared to the ND group. However, there was a significant increase villous/extravillous SOD ratio when treated with melatonin.

The SOD/O_2_^–^ ratio in the maternal blood and the placental villous and extravillous layers in both groups are shown in Table 3. Blood MN cells and placenta villous were lower in T2DM. Melatonin treatment increased the SOD/O_2_^–^ ratio in the T2DM group with values similar to those found in normoglycemic mothers. There was no difference between the groups studied in the SOD/O_2_^–^ ratio in the placental extravillous layer (Table 3).

**TABLE 3:** Superoxide dismutase (SOD) and release of superoxide anion (O2-) rates (SOD/O_2_^–^) in the culture supernatant of MN cells of blood and placenta treated or not by melatonin of diabetic mothers.

Parameters		ND	T2DM
Maternal blood	MLT (−)	10.9 ± 4.7	2.7 ± 1.3*^#^
	MLT (+)	8.8 ± 3.7	11.0 ± 5.7
Placenta villous	MLT (−)	12.4 ± 4.3	4.1 ± 1.7*
	MLT (+)	14.0 ± 6.9	16.5 ± 5.7^#^
Placenta extravillous	MLT (−)	11.5 ± 3.5	11.6 ± 2.4^†^
	MLT (+)	9.6 ± 4.9^†^	7.5 ± 3.5^#†^

*The results represent the mean and standard deviation of 10 samples. MN, Mononuclear cells; MLT, melatonin (MLT).*

P < 0.05 statistical difference (ANOVA test) * comparing the groups considering the same treatment and type of sample; ^#^ comparing untreated cells with cells treated with melatonin, considering the same group and sample; ^†^comparing between villous and extravillous placental layers considering the same treatment and group.

Higher apoptosis rates were found in maternal blood cells from the diabetic group relative to the non-diabetic group. Melatonin treatment reduced the apoptosis rates in this group relative to the ND group. In the placenta villous, the apoptosis rates were lower in the T2DM group irrespective of melatonin stimulation. In both groups, there were no significant differences between the apoptosis indices of MN cells from placenta extravilli (Table 4).

**TABLE 4:** Viability index (%) and apoptosis (%) of MN cells treated by melatonin from maternal blood and placenta from the non-diabetic (ND) and Type II Diabetes Mellitus (T2DM) groups.

MN cells		ND		T2DM	
		Viable	Apoptosis	Viable	Apoptosis
Maternal blood	MLT (−)	78.9 ± 9.1	21.1 ± 8.5	68.6 ± 12.7	41.4 ± 11.0^#^
	MLT (+)	80.4 ± 6.8	19.6 ± 5.7	82.9 ± 4.6*	17.1 ± 3.7*
Placenta villous	MLT (−)	76.5 ± 16.4	33.0 ± 5.8	82.5 ± 6.4	16.1 ± 5.7^#^
	MLT (+)	81.4 ± 9.1	26.1 ± 6.6	78.7 ± 7.0	18.8 ± 5.4^#^
Placenta extravillous	MLT (−)	79.4 ± 5.1	21.7 ± 11.4^†^	73.7 ± 11.0	23.8 ± 8.9^†^
	MLT (+)	78 ± 5.0	22.1 ± 11.4	75.4 ± 10.7	22.8 ± 11.1

*The results represent the mean and standard deviation of 10 samples. MN, Mononuclear cells; MLT, melatonin.*

P < 0.05 statistical difference (ANOVA test) * comparing the groups considering the same treatment and type of sample; ^#^ comparing untreated cells with cells treated with melatonin, considering the same group and sample; ^†^comparing between villous and extravillous placental layers considering the same treatment and group.

## Discussion

Hyperglycemia maternal promotes the production of reactive oxygen species (ROS), resulting in oxidative stress, which can contribute to the proinflammatory environment typical of diabetes (Cvitic et al., 2014) and placental hypervascularization, with alterations in vasculogenesis and VEGF-R1 and R2 receptors (Pietro et al., 2010). In this work, hyperglycemia was able to alter the balance between superoxide anion production and the enzyme superoxide dismutase (SOD) in maternal blood and the placenta of diabetic mothers. These alterations were reflected in the mechanisms of induction of apoptosis and were modulated by the hormone melatonin.

Melatonin assessed in the morning in diabetic mothers had higher levels in maternal blood and lower in placental villi. However, maternal blood and placenta melatonin concentrations of women are variable. Some authors report higher hormone concentration in the blood (Ejaz et al., 2021) and tissue (Lanoix et al., 2012), while others show lower levels (Bouchlariotou et al., 2014) similar to those valours found in blood and placenta in this study and colostrum (Morceli et al., 2013) from diabetic mothers.

The blood MN cells of diabetic mothers presented an increase in superoxide anion, lower release of the enzyme superoxide dismutase, and reduction in the SOD/O_2_^–^ ratio, suggesting alterations in the balance between the prooxidant and antioxidant systems. The free radical generation by mononuclear cells, including the superoxide anion, is an important defense mechanism against infectious diseases (França et al., 2011; Fagundes et al., 2018), although the balance between the pro-and antioxidant mechanisms is also necessary since elevated levels of free radicals cause damage to cells that may result in the activation of cell death pathways (Benirschke, 2000; Maritim et al., 2003; Ferrari et al., 2011; Fernandes et al., 2019).

Various mechanisms have been proposed for the generation of reactive oxygen species. In diabetic patients, glucose oxidation may be the main source of free radicals (França et al., 2008; Ferrari et al., 2011). Hyperglycemia leads to lipid peroxidation by a superoxide-dependent pathway, resulting in the generation and release of free radicals (Rui et al., 2016). Additionally, the interaction of glucose with proteins that promote advanced glycosylation end products contributes to the excess free radical formation (Lappas et al., 2011).

Melatonin increased the release of superoxide in the non-diabetic group and reduced the release of this anion in MN cells of the diabetic group. This reduction affected the SOD/O_2_^–^ ratio, resulting in values similar to those found in the control group.

The melatonin functions in humans are still partially understood. Melatonin can be associated with mononuclear cells, having even been related to the oxidative processes of the organism (França et al., 2008). Melatonin, depending on the dose administered, may have an antioxidative effect, including the scavenging of radicals (França et al., 2008) or pro-oxidative function (Hara et al., 2013; Honorio-França et al., 2013).

Melatonin increases superoxide production in MN phagocytes of non-diabetic mothers but not in the phagocytes of diabetic women. Maternal hyperglycemia alters the functional activity of these cells, and its effects are probably attributed to non-inflammatory processes, with lower superoxide release (Morceli et al., 2013). Inadequate stimulation of the activity of MN cells by melatonin, due to a failure in the prooxidant mechanisms, indicates an antioxidant effect related to diabetes (Reiter et al., 2008). Similar results were reported in diabetes models induced by the diabetogenic drug alloxan (Pawlak et al., 2005; Devi et al., 2008; França et al., 2008).

Alterations in the pro- and antioxidant mechanisms were also shown in the placenta being linked with its layers (extravillous and villous layers). The maternal layer showed a lower concentration of SOD enzyme. The presence of melatonin restored the levels of this enzyme with values similar to those of the control group. In addition, there was an increase in superoxide in the fetal layer and a reduction in the SOD/O_2_ ratio. In the presence of melatonin, there was a reduction in superoxide. The SOD/O_2_ ratio presented values similar to those of the control group, suggesting that in both layers of the placenta, melatonin could reduce oxidative stress, possibly caused by hyperglycemia.

Experimental studies in diabetic pregnancies have associated increased oxidative stress and reduced antioxidant capacity with abnormalities in the structure and function of the placenta (White et al., 2002). Thus, oxidative stress occurs in women with diabetic pregnancy and probably compromises antioxidant defense mechanisms and increases the generation of free radicals (Biri et al., 2009).

As a barrier between the mother and fetus, the placenta targets environmental changes (Radaelli et al., 2003). This organ can alter maternal blood cell and cytokine levels before transferring them to the fetus, and the inflammatory environment influences this mechanism due to hyperglycemia (Hara et al., 2013). Further, there is an impact of maternal diabetes on fetal vascular growth and angiogenesis with placental hypervascularization (Cvitic et al., 2014). These changes in the fetoplacental vasculature in response to maternal diabetes may also imply potential differences in the fetus’s vasculature. Maternal metabolic alterations resulting from hyperglycemia change the uterine environment and may lead to an abnormal fetal growth pattern (Catalano et al., 1999). The key to these alterations is maternal hyperglycemia, with consequent fetal hyperglycemia and hyperinsulinemia, inducing hypoxia, inflammation, and oxidative stress in the intrauterine environment. In this study, changes in oxidative stress found in maternal blood were reflected in the placenta and were probably directed to the fetus.

A relevant result is that the rate of superoxide release (placenta villous/extravillous) was higher in the placenta of T2DM, and melatonin was able to reduce this superoxide rate and increase the rate of SOD. Furthermore, it is known that melatonin can act in the removal of free radicals (Xia et al., 2019) and diabetes control (Sudnikovich et al., 2007) due to its beneficial action associated with the ability to eliminate free radicals and improve antioxidant activity (Sudnikovich et al., 2007; Pandi-Perumal et al., 2008; Xia et al., 2019). In this work, we suggest that melatonin modifies the placental tissue, generating an environment with antioxidant characteristics in the maternal–fetal interface and reinforcing the hypothesis that the placenta acts vicariously to protect the developing fetus.

Although physiological adaptations occur during pregnancy, hyperglycemia may modify these adaptations and determine a higher frequency of complications in pregnancies of diabetic women (Groen et al., 2015). Furthermore, the higher index of superoxide release can alter intracellular events during oxidative metabolism (Fagundes et al., 2012) and plays a key role in the pathogenesis of diabetes (Fagundes et al., 2018), and these changes may increase apoptosis (França et al., 2016; Honorio-França et al., 2016; Honorio-França et al., 2021). In this work, the increased release of superoxide by MN cells in the maternal blood of hyperglycemic mothers associated with reducing the superoxide dismutase enzyme may be associated with high apoptotic rates.

Apoptosis can be considered an essential physiological mechanism through which undesirable tissues and cells can be programmed to be killed in a controlled and tightly regulated manner (Baskić et al., 2006). These alterations are evidenced through morphological changes with DNA damage (Zimmermann et al., 2001). In hyperglycemic mothers, there is an increase in oxidative stress and DNA damage. The type of affected DNA seems to be dependent on the glycemic profile or oxidative stress because the reactive oxygen species resulting from glucose oxidation are more likely to cause DNA damage (Collins et al., 1998), which would explain the increase in apoptosis in the blood cells of mothers with diabetes. It is worth mentioning the important role of the hormone melatonin in this process since this hormone, due to its antioxidant activity, reduced apoptosis indices in the maternal blood of hyperglycemic mothers.

It is evident that in the placental villous layer of diabetic mothers, there was a reduction of the enzyme superoxide dismutase and the apoptotic rates. One of the processes associated with maternal immune tolerance is the apoptosis of T cells that express the Fas ligand in trophoblasts or decidual cells (Guleria and Sayegh, 2007), which confers immune privilege. The apoptosis of maternal immunologic cells expressing cellular surface receptor Fas (CD95) occurs in the placenta/decidua interface. This receptor can regulate the death of various cell types, including β cells of the pancreas, and is associated with the development of type 2 diabetes (Nolsoe et al., 2006).

A previous study showed that diabetic mothers present lower levels of memory T cells in the placental villous layer, associated with lower expression of Fas, suggesting the commitment of apoptosis in MN cells and, probably, in the immunoregulation of the mother-placenta-fetus unit and maternal–fetal tolerance (Queiroz et al., 2019).

The mechanisms by which melatonin influences the cells seem to involve other hormones, cytokines, and specific receptors (Pandi-Perumal et al., 2008; Morais et al., 2019). Also, melatonin increases umbilical and fetal blood flow and antioxidant capacity and contributes to the supply of oxygen and nutrients to increase placental efficiency (Lemley and Vonnahme, 2017).

Melatonin plays a vital role in protecting female reproduction. Lower implantation rates, pregnancy deficiencies, higher incidence of menstrual irregularities, infertility, and miscarriage in women are often associated with changes in melatonin levels in normal uterine and placental tissues. This hormone, well established by the pharmaceutical industry, has been used as a drug in treating several diseases and appears to be effective in treating pre-eclampsia. Thus, considering its lack of toxicity, this study corroborates the potential benefits of melatonin in the reproduction of diabetic mothers and may contribute to its future therapeutic use to minimize the pro-oxidative effects caused by hyperglycemia since melatonin acts, in cells and tissues, against oxidative damage potentially improve maternal and neonatal quality of life.

It should be considered that these data were evaluated during a collection period, which can be considered a limitation of this study. Furthermore, it is necessary to continue investigations focusing on other factors and interactions with other hormones or bioactive components that may be important for the fetus-placental unit.

These data suggest that hyperglycemia could alter the balance between superoxide anion production and superoxide dismutase (SOD) enzyme in maternal blood and the placenta of diabetic mothers. These changes reflected in the apoptosis induction mechanisms and were modulated by the hormone melatonin. These results reinforce the importance of the melatonin hormone in the control of oxidative stress and reduction of apoptosis in the maternal blood and the control of apoptosis in trophoblastic cells, which may probably favor maternal–fetal tolerance and the vascularization of the uteroplacental in diabetic mothers.

## Data Availability Statement

The raw data supporting the conclusions of this article will be made available by the authors, without undue reservation.

## Ethics Statement

The studies involving human participants were reviewed and approved by the Research Ethics Committee of School of Medicine Obstetrics Course, UNESP, Botucatu, SP. The patients/participants provided their written informed consent to participate in this study.

## Author Contributions

ML carried out the assay, participated in the sequence alignment, and drafted the manuscript. DF and AQ participated in collecting samples, carried out the assays, and helped to draft the manuscript. IC participated in the design of the study and helped to draft the manuscript. EF participated in the design of the study and coordination and helped to draft the manuscript. AH-F conceived the study, carried out the assays, participated in its design and coordination, and helped to draft the manuscript. All authors read and approved the final version of the manuscript.

## Conflict of Interest

The authors declare that the research was conducted in the absence of any commercial or financial relationships that could be construed as a potential conflict of interest.

## Publisher’s Note

All claims expressed in this article are solely those of the authors and do not necessarily represent those of their affiliated organizations, or those of the publisher, the editors and the reviewers. Any product that may be evaluated in this article, or claim that may be made by its manufacturer, is not guaranteed or endorsed by the publisher.
